# Predominance and association risk of *Blastocystis hominis* subtype I in colorectal cancer: a case control study

**DOI:** 10.1186/s13027-017-0131-z

**Published:** 2017-04-12

**Authors:** Amr Mohamed Mohamed, Mona Abdelfattah Ahmed, Sabah Abdelghany Ahmed, Sherif Ahmed Al-Semany, Saad Saed Alghamdi, Dina Abdulla Zaglool

**Affiliations:** 1 Laboratory Medicine, Faculty of Applied Medical Sciences, Umm Al-Qura University, Makkah, 7607 Saudi Arabia; 2 Clinical Laboratory Diagnosis, Department of Animal Medicine, Faculty of Veterinary Medicine, Assiut University, Assiut, Egypt; 3 Medical Parasitology, King Abdullah Medical City, Makkah, Saudi Arabia; 4 Parasitology Department, Faculty of Medicine, Ain-Shams University, Cairo, Egypt; 5 Oncology, King Abdullah Medical City, Makkah, Saudi Arabia; 6 Department of Internal Medicine, Medical Oncology, Mansoura University, Mansoura, Egypt; 7 Medical Parasitology, Al-Noor Specialist Hospital, Makkah, Saudi Arabia; 8 Parasitology Department, Faculty of Medicine, Assiut University, Assiut, Egypt

**Keywords:** *Blastocystis hominis*, CRC, Genetic diversity, Subtypes-I, Association risk

## Abstract

**Background:**

*Blastocystis,* a genetically diverse intestinal parasite with controversial pathogenic potential, has increasingly been incriminated for diarrheal illness in immunocompromised individuals including colorectal cancer (CRC) patients. The aim of the current study was to assess the possible association between *Blastocystis* infection and CRC condition in Makkah, Saudi Arabia (KSA).

**Methods:**

Stool samples were collected from 80 non-cancer (NC) and 138 cancer subjects including 74 CRC patients and 64 patients with other cancers outside gastrointestinal tract (COGT). Molecularly confirmed *Blastocystis* isolates were genetically grouped and subtyped using multiplex polymerase chain reaction with restriction fragment length polymorphism (PCR-RFLP) and sequence-tagged site primers-based PCR (PCR-STS), respectively.

**Results:**

*Blastocystis hominis* were confirmed in 29.7, 25 and 15% among CRC, COGT and NC patients, respectively. Obtained *Blastocystis* isolates were initially categorized into 2 groups (A and C), which were subsequently subtyped into 3 different subtypes; subtype-I (38%), subtype-II (44%) and subtype-V (22%). Interestingly, subtype-I was the most predominantly detected subtype (54.5%) among CRC patients with a significant association risk (COR 7.548; 95% CI: 1.629–34.987; *P* = 0.004).

**Conclusion:**

To the best of our knowledge, the current study is the first to provide genetic insights on the prevalence of *Blastocystis hominis* among CRC patients in Makkah, KSA. Moreover, the study suggests for a possible association between subtype-I of *Blastocystis hominis* and CRC, which could indicate a potential influence of Blastocystis on CRC condition. Further studies are required to confirm this association risk and to investigate the possible underlying mechanism of postulated carcinogenic influence of *Blastocystis hominis* subtype-I.

**Electronic supplementary material:**

The online version of this article (doi:10.1186/s13027-017-0131-z) contains supplementary material, which is available to authorized users.

## Background

*Blastocystis* species remains one of the most common intestinal parasites in humans with a prevalence of up to 10% in developed countries, rising to 50–60% in developing countries [1, 2]. It is considered one of the most commonly encountered non-fungal eukaryotic organisms in human fecal samples [3]. Blastocystis is an enteric protozoon found in the intestinal tract of humans and a wide range of animal hosts [4]. Morphologically, *Blastocystis* is a highly polymorphic organism that takes several different forms during its life cycle including vacuolar, cystic, amoeboid, granular, multivacuolar, and avacuolar forms [1, 5].

The pathogenicity of these protozoa is still controversial and inconclusive with non-specific symptoms such as abdominal pain, nausea, vomiting, anorexia, flatulence, weight loss, and acute or chronic diarrhea [6, 7]. Similar to other intestinal parasitism and chronic gastrointestinal illnesses such as irritable bowel syndrome (IBS), *Blastocystis* infection is usually associated with alternate episodes of diarrhea, normal defecation or even constipation. Symptomatic *Blastocystis* infection has been encountered more commonly among patients of IBS as well as other immunocompromised patients [3, 6, 8].

Molecular studies revealed that the parasite is characterized by an extensive genetic diversity in both humans and animals with a worldwide distribution [9–15]. At least 10 subtypes (ST), ST1 to ST10, have been recognized based on the small subunit ribosomal RNA (SSU rRNA) gene sequence [16]. In addition, three novel subtypes (ST11–ST13) have been identified from captive animals in the zoo [17]. At present, only ST1 to ST9 are considered to colonize in humans [7, 17]. This genetic diversity has supported the hypothesis that the variability in symptoms in patients positive for *Blastocystis* could be due to different pathogenic potential among the subtypes [18–21].

Prevalence studies of *Blastocystis* in immunocompromised individuals have been confined to HIV/AIDS patients and there is general lack of information on the prevalence of the organism in other immunocompromised individuals such as colorectal cancer patients. Therefore the current study aimed to assess the possible relationship between *Blastocystis* infection and malignancy with special reference to CRC. The frequency of *Blastocystis* infection among CRC patients in comparison with other cancer and non-cancer patients were investigated. In addition, the study also aimed to assess the association potential of genetically identified subtype(s) of encountered *Blastocystis* infection with CRC in Makkah, KSA. This represents the first study to explore the genetic diversity of encountered *Blastocystis* isolates and to assess their association significance with colorectal cancer in Makkah, KSA.

## Methods

### Study subjects

This was a prospective case control study. A total of 218 stool samples were collected from recruited participants attending King Abdulla Medical city (KAMC), Makkah, KSA during the period extended from April 2013 to March 2015. Recruited participants belonged to two main groups. The first group included recently diagnosed patients with malignancy (138) and referred to as cancer patients (CP) while the second group composed of normal subjects visiting the hospital for routine checkup (80) and referred to as non-cancer patients (NC). The cancer patients were categorized into two subgroups; colorectal cancer group (CRC), which included 74 subject and cancers outside gastrointestinal tract group (COGT), which included 64 patients (14 non hogken lymphoma; 11 malignant neoplasm of bladder; 15 malignant neoplasm of uterine adnexa; 9 malignant neoplasm of larynx and 15 malignant neoplasm of breast). Exclusion criteria included any suspected patient started anti-cancer treatment regime and/or receiving any anti-parasitic medication. Ethical approval for the study was obtained in accordance with the declaration of Helsinki from the Ethics Committee of the Faculty of Applied Medical Sciences, Umm Al-Qura University (AMSEC 10-18-2-2013) and Biomedical Research Ethics committee of King Abdullah Medical City. All investigated patients signed acknowledgment consents to declare their participation agreement.

### Isolation and conventional identification of *Blastocystis*

*Blastocystis* parasites were isolated from suspected stool samples by in vitro cultivation at 37 °C using Jones’ medium supplemented with 10% horse serum for 72 h. [22]. Suspected cultures were then sub-cultured in duplicate using Jones’ medium at 37 °C for 3 additional days. Afterwards, for each suspected isolate, one culture medium was subjected to microscopic examination for conventional identification of suspected *Blastocystis*, while the second culture medium was kept at -20 °C for further molecular studies.

### Molecular identification of isolated Blastocystis

Genomic DNA was isolated from cultures of conventionally identified *Blastocystis* as previously described with few modifications [23]. Briefly, frozen culture media were thawed at room temperature and the suspected *Blastocystis* were harvested by centrifugation at 500 × g for 5 min and washed with sterile phosphate- buffered saline (PBS) (pH 7.4) for 5 times. Obtained cell pellets were then lysed using lysis buffer (20 mM Tris–HCl buffer, pH 8.0, 100 mM NaCl, 25 mM EDTA, pH 8.0) containing 1% SDS and 0.5 mg Proteinase K/ml (Fermentas, USA) and incubated at 55 °C overnight. Genomic DNA was then extracted with phenol/chloroform/ isoamyl alcohol. Extracted DNA was then precipitated in 2 vol of ice-cold ethanol containing 0.3 M sodium acetate (pH 5.2). Obtained DNA pellets were first washed in 70% ice-cold ethanol, and then was resuspended in 50 μl Tris–EDTA (TE) buffer (10 mM Tris, 1 mM EDTA, pH 8.0). DNA concentration of each sample was determined using the BioSpec-nano (Shimadzu Corporation, Japan) and its quality and integrity was tested using the A260/A280 ratio.

Identification of *Blastocystis* isolates was confirmed by molecular amplification of the conserved 1.1 Kbp. Target of SSU rRNA gene using previously described primers Blas-F: (GGA GGT AGT GAC AAT AAA TC) and Blas-R: (ACT AGG AAT TCC TCG TTC ATG) [24]. Amplification protocol was carried out as previously described with some modification [25]. Briefly, 5 μl of template DNA (10 ng/ μl) were used in a total reaction volume of 50 μl. The reaction mix included PCR buffer (20 mmol Tris-HCL (pH 8.4) and 50 mmol KCl), 0.1 mmol each of dNTP (deoxyribonucleotide triphosphate), 1.5 mmol of MgCl2, 50 pmol of each primer, and 1.5 U of HotStar HiFidelity Polymerase (Qiagen). PCR condition consisted of an initial denaturation step at 95 °C for 10 min followed by 35 cycles of denaturation at 95 °C for 1 min, annealing at 53 °C for 30 s, and extension at 72 °C for 1 min.

### Grouping and subtyping of Blastocystis by PCR-RFLP and PCR-STS analysis

Grouping of *Blastocystis* isolates was performed by RFLP analysis of the amplified 1.1 kbp. target of SSU rRNA gene using *SpeI* restriction enzyme (New England BioLabs Inc., MA, USA). *Blastocystis* isolates were grouped according to size of obtained digestion products as previously described [26]. Grouped *Blastocystis* isolates were then subjected to subtyping analysis. For this purpose, seven pairs of sequence-tagged site (STS) previously described primers [9] were used for conducting PCR-STS analysis. Primer sets names and sequences as well as predicted product size are shown in Table 1. PCR-STS protocol was conducted as previously described with little modification [9, 23]. Briefly, 5 μl of template DNA (10 ng/ μl) were used in a total reaction volume of 50 μl. The reaction mix included PCR buffer (20 mmol Tris-HCL (pH 8.4) and 50 mmol KCl), 0.1 mmol each of dNTP (deoxyribonucleotide triphosphate), 1.5 mmol of MgCl2, 25 pmol of each primer, and 1.5 U of HotStar HiFidelity Polymerase (Qiagen). The PCR amplification started with an initial denaturation step at 95 °C for 10 min, followed by 35 cycles including denaturation at 95 °C for 1 min, an annealing at 56 °C for 30 s, and an extension step at 72 °C for 1 min. All PCR amplifications were carried out using Applied Biosystems Veriti Thermal Cycler (ThermoFisher Scientific Inc.) After PCR, 10 μl of the PCR product was mixed with 5 μl dye mixture (0.25% bromophenol blue and 0.25% xylene cyanol in 15% Ficoll type 400) and electrophoresed in 1 μl Tris-acetate-EDTA buffer through a 2% agarose gel containing ethidium bromide (0.5 μg/mL). Bands of the appropriate size were visualized using a Molecular Imager® Gel Doc™ XR System (Bio-Rad Laboratories) according to the manufacturer’s instructions and identified by comparison with a 100-bp DNA ladder (DNA molecular weight marker Promega) using Image Lab version 5 (Bio-Rad Laboratories).

**Table 1:** Different STS primer sets used for differential identification of Blastocystis subtypes along with expected amplified product sizes

STS primer set	GenBank accession no.	Sequences	Product size	Subtype
SB83	AF166086	F-GAAGGACTCTCTGACGATGA R-GTCCAAATGAAAGGCAGC	351	I
SB155	AF166087	F-ATCAGCCTACAATCTCCTC R-ATCGCCACTTCTCCAAT	650	II
SB227	AF166088	F-ATCAGCCTACAATCTCCTC R-ATCGCCACTTCTCCAAT	526	III
SB332	AF166091	FGCATCCAGACTACTATCAACATT R-CCATTTTCAGACAACCACTTA	338	IV
SB340	AY048752	F-TGTTCTTGTGTCTTCTCAGCTC R-TTCTTTCACACTCCCGTCAT	704	V
SB336	AY048751	F-GTGGGTAGAGGAAGGAAAACA R-AGAACAAGTCGATGAAGTGAGAT	317	VI
SB337	AY048750	F-GTCTTTCCCTGTCTATTCTGCA R-AATTCGGTCTGCTTCTTCTG	487	VII

### Statistical analysis

Statistical analysis of the results was performed using SPSS version 16 (SPSS, Chicago, IL). The frequencies of *Blastocystis* infection and/or its subtypes among different groups of investigated patients were assessed using cross-tabulation followed by Chi square (*X*2) test or Fischer’s exact test. A crude odds ratio (COR) with 95% confidence interval (CI) was calculated for frequency analysis as appropriate for assessment of possible association risk. All tests performed were two-sided and *P* value < 0.05 was considered significant.

## Results

### Detection and identification of *Blastocystis* isolates

**Fig. 1:**
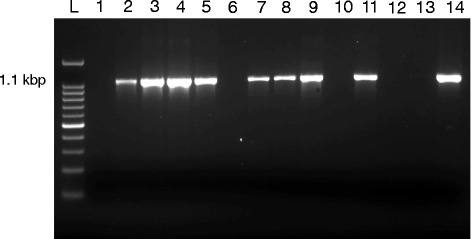
Representative 1% agarose gel showing the amplification of the *Blastocystis*-specific 1.1 kbp target of the SSU of rRNA gene. (Lane L) shows 100 bp DNA ladder; (Lane 1) represents negative control; (Lanes 2,3,4,5,7,8,9,11and 14) represent positive results for *Blastocystis*; (Lanes 6,10,12 and 13) represent negative results for *Blastocystis*

*Blastocystis* was initially identified conventionally by microscopic visualization of *Blastocystis* stages in culture media. Identification was then confirmed genetically by PCR amplification of the conserved Blastocystis hominis-specific 1.1 Kbp. target of the SSU rRNA gene (Fig. 1). Out of a total of 218 fecal samples from suspected patients with gastrointestinal illnesses, *Blastocystis hominis* were identified in 50 (22.9%) samples. This included 22 (29.7%) of CRC patients, 16 (25%) of COGT patients and 12 (15%) of NC patients (Table 2). A significant difference (*P* < 0.05) of *Blastocystis* infection frequency was evident between CP group and NC groups as well as between CRC and NC groups. However, no significant difference (*P* > 0.05) was evident between COGT and NC groups (Table 2). Moreover, results revealed an associated risk (COR 2.153; 95% CI: 1.053-4.417; *P* = 0.044) between *Blastocystis* infection and cancer condition with a higher risk of association (COR 2.397; 95% CI: 1.087 – 5.286; *P* = 0.033) in CRC group but not in the COGT group.

**Table 2:** Frequency of Blastocystis infection among CP including CRC and COGT groups as well as NC group of investigated patients

Investigated groups (no.)		Blastocystis infection			
		Positive cases no. (%)		Negative cases no. (%)	
CP (138)	CRC (74)	38 (27.5)^*a*^	22 (29.7)^b^	100 (72.5)	52 (70.3)
	COGT (64)		16 (25)		48 (75)
NC (80)		12 (15)		68 (85)	
Total (218)		50 (22.9)		168 (77.1)	

Cancer patients (CP); Colorectal cancer (CRC); COGT (Cancer outside gastrointestinal tract); Non cancer (NC)

^*a*^Blastocystis infection in overall cancer group as compared to NC group (*P* = 0.044)

^*b*^Blastocystis infection in CRC group as compared to NC group (*P* = 0.033)

### Genetic grouping and subtyping of *Blastocystis* isolates

**Fig. 2:**
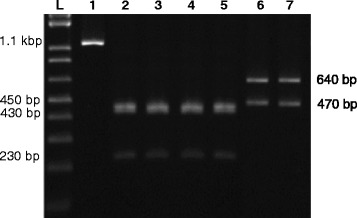
Representative 1% agarose gel showing *Blastocystis* grouping based on RFLP-PCR of the *Blastocystis*-specific 1.1 kbp target of the SSU of rRNA gene. (Lane L) shows 1 Kbp. DNA ladder; (Lane 1) shows undigested 1.1 kbp target of *Blastocystis.* (Lanes 2–5) show 230 bp, 430 bp and 450 bp digestion products corresponding to *Blastocystis* group A. (Lanes 6 and 7) show 470 bp and 640 bp digestion products corresponding to *Blastocystis* group C

**Fig. 3:**
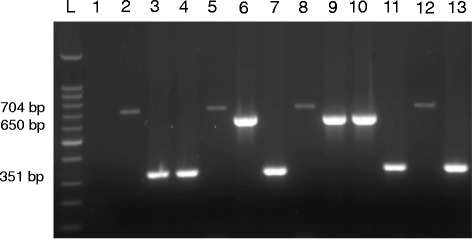
Representative 1% agarose gel showing *Blastocystis* subtyping based on PCR-STS analysis. (Lane L) shows 100 bp DNA ladder; (Lane 1) represents negative control. (Lanes 2,5,8 and 12) show 704 bp PCR products and represent *Blastocystis* subtype-V. (Lanes 3,4,7,11 and 13) show 351 bp PCR products and represent *Blastocystis* subtype-I. (Lanes 6,9 and 10) show 650 bp PCR products and represent *Blastocystis* subtype-II

Based on RFLP analysis of the amplified 1.1 Kbp target of the SSU rRNA gene, 2 *Blastocystis* groups were reported; group A (230 bp., 430 bp. and 450 bp.) and group C (470 bp. and 650 bp.) (Fig. 2). Accordingly, 39 isolates of obtained *Blastocystis hominis* from suspected patients were categorized as group A while the rest 11 isolates were categorized as group C. further genotypic analysis of recovered *Blastocystis hominis* based on the predicted size of obtained amplicons after PCR-STS assay resulted in subtyping of group A isolates into 2 different subtypes (I and II) and that of group C as only one subtype (V) (Fig. 3). Overall, 40% of recovered *Blastocystis hominis* were identified as subtype II, 38% as subtype I, and 22% were identified as subtype V. In relation to type of patients, subtype I was predominant (44.7%) in cancer patients while subtype II was predominant (58.3%) among non-cancer patients. Among cancer patients, subtype I was the predominant subtype (54.5%) among CRC patients, while subtype II was predominant (43.7%) among COGT patients (Table 3). Subtype I showed significant higher frequency (*P* < 0.05) in CRC group as compared to other recovered subtypes in the same group. With regard to frequency significance among different groups, subtype I showed significant higher frequency (*P* < 0.05) in CP as compared to NC group. On the other hand, frequency variation between different cancer patients groups revealed significant higher frequency (*P* < 0.05) of subtype I in CRC as compared to COGT group (Table 3). Interestingly, an association risk between *Blastocystis* subtype-I and cancer condition was evident (COR 5.479; 95% CI: 1.232-24.374; *P* = 0.013) with a greater risk of association (COR 7.548; 95% CI: 1.629 – 34.987; *P* = 0.004) in CRC group.

**Table 3:** Frequency of different *Blastocystis* subtypes isolated from CP including CRC and COGT groups and NC group of investigated patients

Investigated groups (n)		Recovered subtypes of Blastocystis no. (%)					
		I		II		V	
CP (38)	CRC (22)	17 (44.7) ^*a*^	12 (54.5) ^*b, c, d, e*^	13 (34.2)	6 (27.3)	8 (21.1)	4 (18.2)
	COGT (16)		5 (31.3)		7 (43.7)		4 (25)
NC (12)		2 (16.7)		7 (58.3)		3 (25)	
Total (50)		19 (38)		20 (40)		11 (22)	

Cancer patients (CP); Colorectal cancer (CRC); COGT (Cancer outside gastrointestinal tract); Non cancer (NC)

^*a*^subtype I in CP group against subtype I in NC group (*P* = 0.013)

^*b*^subtype I in CRC group against subtype I in NC group (*P* = 0.004)

^*c*^subtype I in CRC group against subtype I in COGT group (*P* = 0.024)

^*d*^subtype I in CRC group against subtype II in the same group (*P* = 0.019)

^*e*^subtype I in CRC group against subtype V in the same group (*P* = 0.018)

All raw data of the current work were made available as an additional file (Additional file 1).

## Discussion

After dietary factors and tobacco smoke, infectious diseases represent the third leading cause of cancer worldwide. The International Agency on Research of Cancer (IARC) has estimated that 16% of cancer worldwide is initiated by infectious agents including parasites [27]. With regard to relation between parasitic infection and cancerous conditions, few studies investigated the association between *Blastocystis hominis* infection and colorectal cancer. The recently postulated potential carcinogenic effect of *Blastocystis hominis* infection in human host especially in colorectal cancer patients [28, 29] signifies the need for screening of colorectal cancer patients for *Blastocystis hominis* infection. Therefore the current study aimed to investigate the association risk of *Blastocystis hominis* infection and its subtypes in relation to CRC patients of Makkah region, KSA.

The over all rate of *Blastocystis* infection as revealed in the current study was 22.9%. The current finding is comparable to previously reported frequencies in several countries including Saudi Arabia (17.5%) [30], Malaysia (25.7%) [31], Jordan (25%) [32], Egypt (31%) [33], 25.78% in Venezuela [34] and 22.9% in Argentina [35]. On the other hand, significantly lower infection rates ranged between 9 and 12% were recently reported in Saudi Arabia [36, 37]. The currently recorded higher infection rate could be attributed to the type of targeted patients in the comparable studies where the patients were predominantly immune-competent. Interestingly, the currently recorded rate of *Blastocystis hominis* infection among cancer patients is apparently higher than figures (7.7–13%) previously recorded among other cancer patients [23, 38, 39]. With regard to type of patients, current study revealed significant higher frequency (*P* = 0.044) of *Blastocystis* infection in CP group as compared to NC group. This finding might support the association risk of the parasite with immunocompromised condition [23] and denotes that *Blastocystis* infection is not rare and should be looked for routinely in immunocompromised patients. However, with regard to type of cancer, the current study revealed significant (*P* = 0.033) higher frequency of *Blastocystis* infection only in CRC and not COGT group as compared to NC group. These findings contradict the notion of association between *Blastocystis* infection and immunocompromised conditions, yet denote that *Blastocystis* infection is more likely associated with those immunocompromised conditions with gastrointestinal affections as CRC. This likely association was statistically validated in the current study (COR 2.397; 95% CI: 1.087–5.286; *P* = 0.033).

Genetic diversity of *Blastocystis hominis* and its worldwide distribution has been evidenced [9–13]. Genotyping of the parasite has received great attention lately in a trial to link the different pathogenic behaviors of the parasite to its different subtypes. Based on the RFLP analysis of the small subunit ribosomal RNA gene, *Blastocystis hominis* are classified into four groups as previously described [26]. In the current study PCR-RFLP analysis revealed that most of the obtained isolates from suspected patients with gastrointestinal symptoms belonged to group A (78%) while the rest of the isolates were found belonging to group C (22%). Further subtyping of obtained isolates was carried out using PCR-STS assay as previously described [9]. Dissimilar to previous studies, which usually reveal the predominance of one subtype among investigated local population [22, 24, 40], the current study revealed the presence of 3 different subtypes (I, II and V) among investigated patients. The detection of multiple subtypes could be due to the exceptional setting of the study. Makkah, where the current study was conducted, is a unique place in Saudi Arabia and the entire world. Annually, it receives more than three million pilgrims during the pilgrimage season in addition to several other million visitors during the whole year from allover the world [41, 42]. This could have contributed to the acquisition of different *Blastocystis hominis* subtypes from different worldwide settings. In disagreement with previous studies, which had shown the predominance of subtype III among patients with chronic gastrointestinal illness in Malaysia [22], Singapore [24], Egypt [43], Turkey [44], USA [45] and Iran [46], interestingly, the current study have showed the predominance of subtype I and II (38 and 40%, respectively) among targeted patients in Makkah. However, the current finding was in agreement with a previous study in central Thailand, where two subtypes (ST1 and ST2) were found predominant among schoolchildren of a rural community [47]. In the current study subtype II, most likely the one that is non-pathogenic [19], was the most predominantly detected subtype among investigated patients. On the other hand, subtype I, the second most predominately detected subtype among investigated patients, is believed to be one of the known pathogenic subtypes that were implicated in several human diseases and believed to be of animal origin with a zoonotic potential [9, 13]. Evaluation of current study results revealed significant predominance (*P* = 0.019 and *P* = 0.018, respectively) of subtype I as compared to other recovered subtypes (II and V, respectively) in CRC patients. Interestingly, the significant predominance of subtype I among CP group as compared to NC patients included highly significant predominance (*P* = 0.004) in CRC group and not COGT group. Moreover, a strong association risk (COR 7.548; 95% CI: 1.629–34.987; *P* = 0.004) was evident between *Blastocystis hominis* subtype-I infection and CRC condition. This interesting finding supports the postulated carcinogenic effect of certain *Blastocystis hominis* subtypes and their possible influence on colorectal cancer. Recently, in vitro studies documented the ability of *Blastocystis hominis* to induce the growth of colorectal cancer cell lines via inhibiting the apoptotic effect of colon cancer cells. Furthermore, isolated antigens of *Blastocystis hominis* isolates were shown to promote the proliferation of cancer cells via down-regulation of host immune cellular responses [28, 29].

Growing data has evidenced the zoonotic potential of *Blastocystis* spp., where comparable genetic sequences were documented in a number of studies between different *Blastocystis* spp. isolated from both human and animals [48–50]. Moreover, some specific *Blastocystis hominis* subtypes were identified in both humans and animals. Subtype I is a common subtypes that had been identified in both human and a wide range of animal species including pigs, horses, monkeys, cattle, rodents, chickens, quails, and pheasants [10, 50, 51]. Other subtypes were also implicated in both human and animal infections with a potential zoonotic ability like subtype V, the least predominant subtype encountered in the current study, which was reported in both human and dogs from the same sitting in Thailand [49]. In general, it was reported that population closely associated with animals has a higher prevalence of blastocystosis when compared with those not associated with animals [52]. With regard to the current study, the association risk between investigated suspected patients and animals was not inspected. However, it worth mentioning that many residents at Makkah region have some sort of association with animals particularly sheep, goat and camels. Moreover, drinking unpasteurized or even raw milk from dairy animals, a trend that is widely practiced in the region is a potential risk factor for disease transmission from animals to human [3]. Nevertheless, further epidemiologic studies need to be conducted to provide additional evidences to confirm the postulated role of zoonotic transmission of the disease to human population in Makkah, KSA.

## Conclusions

The current study is the first to provide genetic insights on the prevalence of *Blastocystis hominis* among CRC patients in Makkah, KSA. Interestingly, the current results suggested a possible association between *Blastocystis hominis* subtype-I and CRC condition, which postulate a potential influence of this pathogen on carcinogenesis of CRC. In deed, these findings need to be confirmed via further controlled epidemiologic and topographic investigations to confirm the proposed association risk and to reveal other possible risk factors that could contribute to the condition. In addition, further studies are required to explore the underlying mechanism of the postulated carcinogenic influence of this pathogen.

## Additional file

Additional file 1: Raw results data of different patient groups. Description of data: The original numbers and information of different groups of investigated patients along with the raw data of the obtained results. (XLSX 50 kb)
